# Aragonite Precipitation by “*Proto-Polyps*” in Coral Cell Cultures

**DOI:** 10.1371/journal.pone.0035049

**Published:** 2012-04-13

**Authors:** Tali Mass, Jeana L. Drake, Liti Haramaty, Yair Rosenthal, Oscar M. E. Schofield, Robert M. Sherrell, Paul G. Falkowski

**Affiliations:** 1 Institute of Marine and Coastal Sciences, Rutgers, State University of New Jersey, New Brunswick, New Jersey, United States of America; 2 Department of Earth and Planetary Sciences, Rutgers, State University of New Jersey, Piscataway, New Jersey, United States of America; Heriot-Watt University, United Kingdom

## Abstract

The mechanisms of coral calcification at the molecular, cellular and tissue levels are poorly understood. In this study, we examine calcium carbonate precipitation using novel coral tissue cultures that aggregate to form “*proto-polyps*”. Our goal is to establish an experimental system in which calcification is facilitated at the cellular level, while simultaneously allowing *in vitro* manipulations of the calcifying fluid. This novel coral culturing technique enables us to study the mechanisms of biomineralization and their implications for geochemical proxies. Viable cell cultures of the hermatypic, zooxanthellate coral, *Stylophora pistillata*, have been maintained for 6 to 8 weeks. Using an enriched seawater medium with aragonite saturation state similar to open ocean surface waters (Ω_arag_∼4), the primary cell cultures assemble into “*proto-polyps*” which form an extracellular organic matrix (ECM) and precipitate aragonite crystals. These extracellular aragonite crystals, about 10 µm in length, are formed on the external face of the *proto-polyps* and are identified by their distinctive elongated crystallography and X-ray diffraction pattern. The precipitation of aragonite is independent of photosynthesis by the zooxanthellae, and does not occur in control experiments lacking coral cells or when the coral cells are poisoned with sodium azide. Our results demonstrate that *proto-polyps*, aggregated from primary coral tissue culture, function (from a biomineralization perspective) similarly to whole corals. This approach provides a novel tool for investigating the biophysical mechanism of calcification in these organisms.

## Introduction

The potential effects of global warming and ocean acidification on coral calcification have received significant attention in recent years [1]–[2]. However, despite the broad interest in coral calcification [3]–[6] and the potential for climate-driven adverse effects, the actual calcification mechanisms are still poorly understood at the cellular level. A lack of mechanistic understanding of processes that lead to and control calcification limits our ability to predict corals' response to increasing atmospheric CO_2_ [6]. Thus, if we are to truly predict the effects of increasing atmospheric pCO_2_, it is of critical importance to understand these mechanisms. This will also provide a stronger basis for interpreting geochemical proxies in corals.

Corals (phylum, Cnidaria) belong to one of the oldest invertebrate phyla. They are the earliest metazoans that possess an organized body structure. Their body plan consists of two cell layers: an ectoderm and an endoderm, separated by the mesoglea, which is a non-cellular gelatinous matrix [7]. In symbiotic corals, the endoderm hosts unicellular algae of the genus *Symbiodinium*. These tissues are mechanically anchored to the skeleton by desmocytes [8]. The aboral ectoderm in contact with the skeleton, referred to as the calicoblastic epithelium, is involved in the extracellular production of the aragonite (orthorhombic CaCO_3_) coral skeleton under its apical membrane [9]. The sub-calicoblastic space between the skeleton and the calicoblastic epithelium, containing the “calcifying fluid” in which new skeletal material is precipitated, does not exceed a few nanometers [3]. Accessing this extremely small space is very difficult and has prevented detailed physiological studies of sub-calicoblastic chemical variability. The lack of mechanistic understanding of the calcification process also constrains our ability to interpret geochemical proxies in corals (e.g., δ^11^B for reconstructing seawater pH) using calibrations based solely on correlative geochemical signatures of intact living corals [10]–[12].

Previous studies that attempted to quantify the *in vivo* chemistry at the cellular level rely on microsensor pH measurements within the calcifying fluid or fluorescent dyes that give information about pH and/or calcium concentrations [13], [14]. Using microelectrodes, Al-Horani et al. [10] found, in the coral *Galaxea fascicularis*, large shifts in pH from 9.3 to 7.5 under light and dark conditions, respectively. Similarly, higher pH in the calcifying fluid than in the ambient seawater was reported in the coral *Stylophora pistillata* based on a pH-sensitive fluorescent probe (SNARF-1) [14]. The diurnal pH shift reported in the latter study was lower under comparable light and dark conditions (from ∼8.7 to ∼8.4). However, given that these two studies were conducted on different coral species and with different methodologies, identical pH values should not be expected. Based on their pH measurements, Venn et al. [14] assumed a very high alkalinity for the calcifying fluid (>5000 µmol/kg) and estimated the aragonite saturation state in the sub-calicoblastic space (Ω_arag_) to be ∼20 in the light and ∼11 in the dark. Further support for elevated pH below the calcifying tissue of corals comes from boron isotope measurements of coral skeletons [10]–[12]. These studies suggest that the pH in the calcifying fluid is apparently about 0.4 pH units higher than that of ambient seawater, which could indicate the regulation of pH in the calcifying fluid by the coral. Boron isotopic composition, recorded in the skeleton of the tropical coral *Cladocora caepetos* [15], suggests that corals may adjust the pH of the calcifying fluid by up to ∼1.4 pH units, depending on ambient seawater pH. If true, corals could maintain a similarly high Ω_arag_ over a range of environmental conditions. This observation raises an apparent paradox: if corals can regulate their internal pH, why would they be vulnerable to ocean acidification? A comprehensive answer to this question requires manipulation of the chemistry of calcifying fluids independent of ambient conditions.

The presence of an extracellular organic matrix (ECM) in coral skeletons is well documented [9], [15], [16]. Muscatine and Cernichiari [17] showed that carbon fixed by *Symbiodinium* is transferred to the host and incorporated into ECM. Additionally, isotopically labeled dissolved free amino acids in seawater and in particulate food sources have been shown to accumulate as an organic component in coral skeletons [18]. Skeletal ECM is rich in aspartic and glutamic acid leading to the hypothesis that acidic amino acids are important in the calcification process [19]; however, the sequences of proteins involved in calcification and pathways leading to their accumulation in the calcifying space remain to be determined.

In principle, *in vitro* investigations of coral calcification can be studied at the cellular level because of the simplicity of the cnidarian organization and the small number of cell types. Helman et al. [20] previously reported a primary coral cell culture method that can potentially be used to analyze the mechanism of calcification in these organisms. In that study, the authors showed that the primary cell culture exhibits the fundamental processes involved in coral calcification, including production of extracellular matrix, skeletal organic matrix and evidence for extracellular calcification [20]. However, if this method is to be used as a model system for studying the calcification mechanism in corals, it is necessary to demonstrate the feasibility of dissociated coral cells to re-aggregate and to form a functional calcifying organism. Likewise, it is critical to show that skeletal crystals generated by this system are comparable to those produced *in vivo*.

In this paper, we present the first evidence that primary cell cultures of *Stylophora pistillata*, a well-studied species, re-aggregate to form functional organized cell culture (“*proto-polyps*”), which produces an extracellular organic matrix (ECM). Moreover, we demonstrate that these *proto-polyps* produce extracellular aragonite crystals. This system will provide a critical tool for studying calcification mechanisms through *in vitro* manipulations.

## Materials and Methods

### Cell Cultures

Coral fragments from the zooxanthellate coral, *Stylophora pistillata*, were obtained from nubbins growing in an 800 L, custom-designed aquarium as described previously [21]. Cell cultures were prepared following previously published procedures, with small modifications [20]. Briefly, small fragments of coral were excised from parent colonies and incubated for 3 to 5 h with gentle shaking in calcium-free artificial seawater supplemented with a 3% antibiotics–antimycotics and 20 µg ml^−1^ chloramphenicol solution (GIBCO). Fragments were then transferred to 35×10 mm Primaria culture dishes containing 3 mL of culture medium. The medium was prepared in two steps:

- Dulbecco's Modified Eagle Medium (DMEM) with no glucose (Invitrogen) supplemented with the following major seawater ions: 0.35 g l^−1^ KCl, 1.1 g l^−1^ CaCl_2_, 1 g l^−1^ MgSO_4_ 7H_2_O, 18.1 g l^−1^ NaCl, 0.052 g l^−1^ taurine and 25 mM HEPES buffer [22].
- A mixture of artificial seawater (Instant Ocean sea salt, Aquarium Systems 34 p.s.u.), 12.5% modified DMEM (from step I), 20 µg/mL aspartic acid, 2% heat-inactivated FBS (Invitrogen),1% antibiotic–antimycotics solution (GIBCO), 0.1 µM glucose, and 50 µg ml^−1^ L-ascorbic acid.

Cultures were exposed to the same atmospheric conditions as in the aquarium. Therefore, in order to avoid evaporation of the media and to keep the growth conditions similar to that in the aquarium, the cultures were maintained in an environmental growth chamber (Percival scientific, INC, USA) on a 12∶12 h light∶dark cycle at 26°C and 80% humidity, and the media was replaced every 7 days. The carbonate alkalinity of the final medium solution (without the HEPES buffer) was 2215 µmol kg^−1^, as measured by Gran titration [23]. We note that within the analytical error of the method the titration of the artificial seawater only yielded similar carbonate alkalinity to that determined on whole media, suggesting a minimal effect of the organic components. The pH of the medium was maintained at 8.0±0.1 by the HEPES buffer. We calculated Ω_arag_ to be 3.7 using an online program that calculates *in situ* CO_2_ conditions [24]. These conditions mimic open ocean surface waters and the carbonate saturation state in our aquaria. After 48 h, the coral skeleton was removed from the plate, and the medium with the detached cells was centrifuged (8500× g for 10 min). The pellet was re-suspended in fresh media and filtered through 20 µm nylon mesh to remove cell aggregates and debris. In order to rule out the possibility that observed aragonite crystals were formed by inorganic precipitation in the saturated culture medium the following controls were set: (1) a plate with medium but no culture cells; and (2) a primary cell culture treated with 15 mM sodium azide, that through respiratory inhibition, prevents reduction of O_2_ to water by the heme cofactor of cytochrome oxidase in mitochondria

Chlorophyll *a* concentrations were measured by the method of Jeffrey and Humphrey [22] on a DW2000 spectrophotometer after culture samples had been extracted in 90% acetone overnight at −20°C. A rapid assessment of photosynthetic activity of zooxanthellae was determined by use of a Fluorescence Induction and Relaxation (FIRe) fluorometer [25], [26].

### Microscopy Imaging

Microscopy imaging was carried out with an inverted IX71 epifluorescent microscope (Olympus) and a Zeiss LSM 710 confocal microscope. For confocal images, cells were grown on glass bottom dishes (WillCo-Dish). Excitation wavelengths at 490, 488 nm with emission wavelengths at 510, 667 nm were used for green fluorescence proteins (GFP) and chlorophyll fluorescence measurements, respectively.

For field emission scanning electron microscopy (FE-SEM) and energy-dispersive X-ray spectrometry (EDS), cells were grown on Millicell cell culture inserts in 6-well tissue culture plates (Millipore). These cultures were fixed for 2 h with 2% glutaraldehyde in 0.05 M phosphate buffer (pH 7). Some of the samples were gently washed with distilled water and the rest were incubated in 1 M NaOH at 90°C for 20 minutes to denature cellular membranes, followed by dehydration with an ascending ethanol series (50–100%) and critical point drying with liquid CO_2_. All samples were then coated with gold and platinum and observed on a Zeiss Sigma FE-SEM equipped with Gemini column and Oxford Instruments with an IncaPenta FET-X3 detector.

### DNA Extraction PCR Amplification and Sequencing

Total genomic DNA of the cell culture was extracted from harvested cells using a blood and cell culture DNA mini kit (Qiagen # 13323). *S. pistillata* mitochondrial 16S rDNA and cytochrome oxidase subunit I (COI) were amplified using the primers LCOant 5′- TTT TCY ACT AAT CAT AAA GAT AT-3′ and COIantr 5′- GCC CAC ACA ATA AAG CCC AAT AYY CCA AT-3′ [27]. *Symbiodinium* sp.18S rDNA was amplified using the algae-specific primers ss5z (an equimolar mixture of 5′-GCAGTTATAATTTATTTGATGGTCACTGCTAC-3′ and 5′-GCAGTTATAATTTATTTGATGGTTGCTGCTAC-3′) and the complementary primer ss3z (5′-AGCACTGCGTCAGTCCGAATAATTCACCGG-3′) [16].

All PCR reactions contained 0.1–0.4 µg of template DNA, 10 mM total dNTP, 1× REDTaq reaction buffer, 0.1–0.5 µM of each primer and 0.05 unit µL^−1^ of REDTaq polymerase (sigma #D4309) in a total volume of 25–50 µL. Amplifications were performed using a Perkin Elmer-Cetus 480 Thermal cycler with the following thermal profile for the *S. pistillata* and algae-specific primers respectively: 40 cycles of 30 sec at 94°C, 30 sec at 40°C, 90 sec at 72°C, and 35 cycles of 1 min at 94°C, 2 min at 55°C, 3 min at 72°C. PCR products were cloned using the TOPO TA cloning kit (Invitrogen) and transformed into *Escherichia coli* (Top10). Plasmids were purified by the QIAprep spin miniprep kit (Qiagen) and sequenced by Genwiz sequencing service (http://www.genewiz.com/).

### Characterization of CaCO_3_ and ECM

Aragonite mineralogy was confirmed for 7–10 days old culture by X-ray diffraction (XRD) on a Bruker/Siemens Hi-Star detector. Contents of 5–10 wells of each treatment (see above) were scraped with a rubber policeman, combined, and centrifuged. The pellet was rinsed with Milli-Q deionized water and then resuspended in 1 M NaOH and heated to 90°C for one hour. Samples were again centrifuged and the NaOH-insoluble pellet was rinsed with Milli-Q water, dried at 60°C, and stored until analysis. Additionally, skeleton samples of the mother colony were bleached in 1% commercial bleach at room temperature for 4 h, rinsed extensively, dried, and ground to a fine powder with mortar and pestle made of agate. The powder was soaked again for 4 hours in 1% bleach, rinsed extensively, and dried. Samples were mounted onto borosilicate capillary tubes and analyzed by XRD. We added <1 mg LaB_6_ (NIST SRM-660a), a calibration standard, to the culture samples to confirm accuracy of peak identification; this is common for very small samples (i.e.; <1% of the aragonite mass used in mother colony skeleton analyses). Resulting spectra were processed in GADDS software (Bruker AXS).

Amino acid composition of ECM proteins was determined by high performance liquid chromatography (HPLC) after acid hydrolysis of proteins extracted from decalcified skeletons of *S. pistillata* and from NaOH-insoluble culture ECM. Briefly, skeletons were cleaned as described above for XRD, then subsamples of clean skeleton powder and NaOH-treated culture pellet were decalcified in 1N HCl for four hours. The solutions were neutralized and water-soluble and insoluble proteins were separated by centrifugation. Soluble proteins in the supernatant were concentrated by centrifugal filtration (3500× g) on Amicon Ultra filters (Millipore, 3 kDa cut-off), and both solubility fractions were then lyophilized. Dried organic matrix samples were hydrolyzed in 6 N hydrochloric acid at 110°C for 18 hours and then neutralized with NaOH. Subsamples were analyzed according to the modified methods of Mopper and Lindroth [28]. Hydrolyzed amino acids were combined with o-phthaldialdehyde/N-acetyl-L-cysteine in 0.8 M borate buffer (1∶3) and allowed to react at room temperature for 2 min. The derivatization solution was run on a Shimadzu HPLC fitted with an ODS Rexchrom column (Regis, 5 µm) and sodium acetate/methanol mobile phase.

## Results and Discussion

### Self-assembly of *proto-polyps*

**Figure 1:**
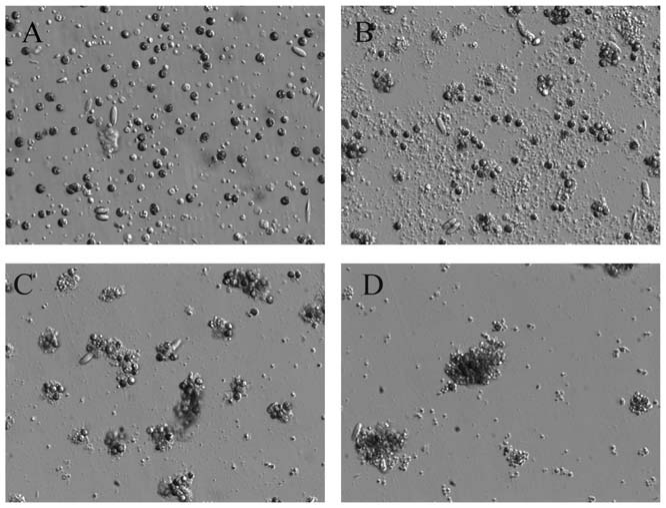
Relief contrast images of coral cell cultures. (A) Relief contrast of individual cells in *Stylophora pistillata* primary cell culture at T_0._ Assembly of *proto-polyps* after (B) 40 h (C) 50 h and (D) 73 h in culture (magnification 20×).

Within 2 days of culture initiation, *S. pistillata* tissue, which had been pre-incubated for 3 to 5 h in calcium-free seawater, spontaneously dissociated from the skeleton into separate, discrete cells. Dissociated cells consisting of a mixture of cell types including free *Symbiodinium* sp. and individual endoderm and ectoderm cells (Fig. 1A), were placed in the culture dish. These cells do not appear to morphologically dedifferentiate (*i.e*., they do not form stem cells), however, transferring the coral fragment to the culture medium suppressed photosynthetic activity in the *Symbiodinium* sp. After 24 hours, photosynthetic efficiency, measured as F_v_/F_m_, was undeterminable compared with intact coral fragments (∼0.50). It should be noted, however, that the chlorophyll concentration did not change during this time period. The observed inhibition of photosynthesis could have been the result of the glucose added to the culture medium that was utilized as an exogenous organic substrate (in lieu of photosynthetic fixation) and subsequently respired by the *Symbiodinium* sp. [20], [29].

The presence of coral cells and *Symbiodinium* sp. in the cultures was confirmed by polymerase chain reaction (PCR) using anthozoan- and *Symbiodinium-*specific primers and blasting the PCR-derived sequences against the National Center for Biotechnology Information nucleotide database (www.ncbi.nlm.nih.gov) (Fig. S1).

**Figure 2:**
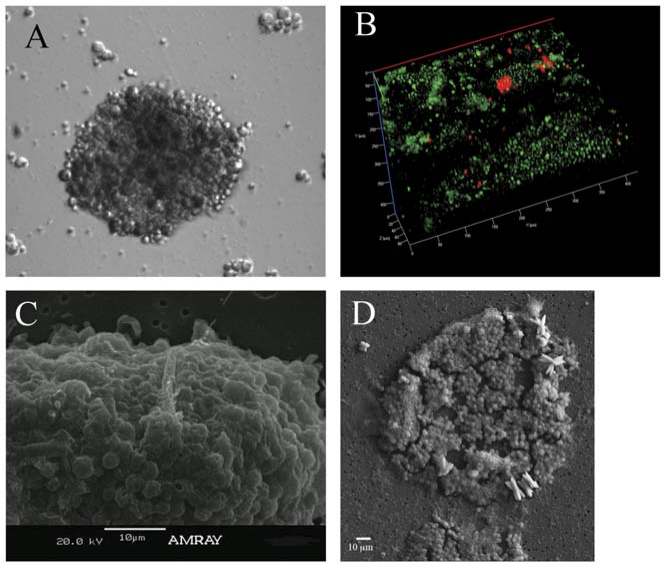
Relief contrast, confocal and SEM images of *proto-polyp*. (A) 72 h old *proto-polyp* (magnification 40×). (B) Z-stack of a *proto-polyp* at 12 d. The *Symbiodinium* sp. cells are seen by chlorophyll florescence and animal cells are revealed by GFP fluorescence. (C) SEM of 12 d old *proto-polyp* cell organization, and (D) extracellular precipitation of aragonite crystals associated with *proto-polyp*.

Within 48 h, individual cells in the culture assembled into organized cell clusters, consisting of 3 layers (Fig. 1B–D, Figs. S2, S3). The viability of these “*proto-polyps*” remained >80% over a period of ∼5 weeks, as was quantitatively assessed by using Sytox green [20], after which microbial contamination became unavoidable. Therefore, all calcification and metabolic measurements were preformed on 5 to 10 day-old uncontaminated cultures. Cells adhered to the Primaria dish substratum through ECM that mediated cell–cell, as well as, cell–substratum adhesion. In addition, assembly of larger spherical aggregations ∼80 µm thick, which detached from the substratum, was observed (Fig. 2A). Cell cultures treated with 15 mM sodium azide did not aggregate, ruling out the possibility of spontaneous aggregation.

Aggregates incorporated all individual cell types (ectoderm or endoderm cells, *Symbiodinium* sp., and nematocysts). *Symbiodinium* sp. cells, as indicated by chlorophyll florescence, were located in the middle part of the aggregation (20–50 µm). In contrast, animal cells, as indicated by GFP fluorescence, were located throughout the aggregate (Fig. 2B, S3). Associated with all aggregates, we observed the formation of aragonite crystals (Fig. 2D).

### Aragonite precipitation

**Figure 3:**
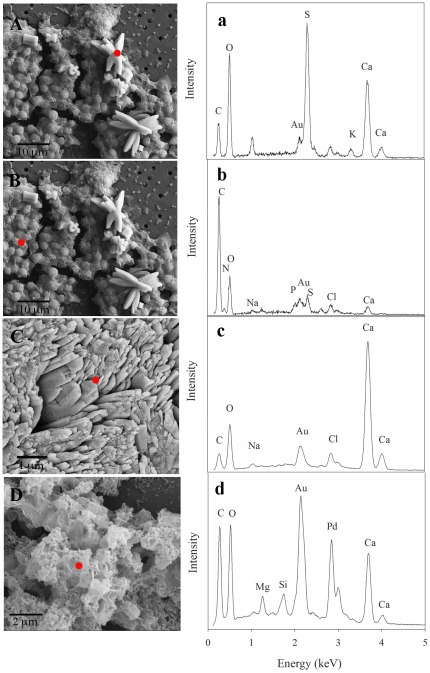
SEM images and elemental composition. SEM images of typical columnar aggregates of aragonite crystals, elongated along the c axis, formed in the dorsal surface of the cell culture (A–B) and in the *S. pistillata* skeleton (C). In both aggregates one can see the orthorhombic structure, characteristic of aragonite crystals. (D) ECM of a *proto-polyp* after cells removed by 1 M NaOH. Red circle on the SEM image indicates the sample point of the EDS. Energy-dispersive X-ray spectrum showing calcium carbonate composition of the aragonite crystal in (a) *proto-polyp*, in (c) coral skeleton and (d) in the ECM scaffold while (b) the composition in the cells is C, O, N and P. The Au and Pd peaks are from the gold coating.

Relatively large, extracellular crystals were detected after 10 days in cell culture, on the surfaces of both adherent and non-adherent *proto-polyps* (Fig. 3A). Crystal growth was not observed on individual cells. Crystals on *proto-polyps* formed distinct flower-shaped bundles, originating from the upper surface facing the media, and were attached to ECM (Fig. 3A). Once the animal's cells were removed from the proto-polyp by 1 M NaOH, the structure of the ECM was revealed (Fig. 3D). In contrast, no calcium carbonate crystals were detected by XRD or EDS in the control treatments, treatments made of culture medium without cells, or with addition of 15 mM sodium azide to healthy cultures.

**Figure 4:**
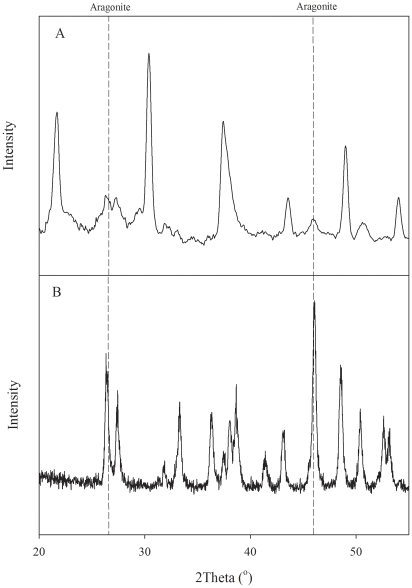
Aragonite mineralogy. X-ray powder diffraction pattern of (A) tissue culture sample and (B) and of a powdered mother colony skeleton showing the characteristic diffraction peaks of aragonite. Composite minerals were determined by peak matching of XRD data in Jade software (MDI Products, Inc.). Peaks at 26.3 and 45.9 degree are characteristic aragonite peaks. Peaks at 21.6, 30.4, 37.4, 43.5, 49, and 54 degrees are due to LaB_6_, added to ensure correct peak identification of very small samples (C).

The elongated shape and morphology of crystals are similar to aragonite crystals observed in the septa of the *S. pistillata* mother colony (Fig. 3C), which are one of the primary sites of CaCO_3_ precipitation [30]. Indeed, XRD analysis of these crystals confirmed that the calcium carbonate polymorph precipitated in our healthy coral cell cultures was aragonite (Fig. 4). Aragonite crystals formed in cell cultures were about 10 µm long. In comparison, aragonite crystals from the mother colony of *S. pistillata*, growing in the in-house aquarium, were smaller than those produced *in vitro* despite the similarity in saturation levels (Fig. 3). Previous studies describe similar morphology and orientation for aragonite crystals at active growing regions of corals as those observed in our tissue cultures [31], [32].

Chemical analysis by energy-dispersive X-ray spectroscopy (EDS) confirmed that crystals observed were, indeed, calcium carbonate. A typical X-ray spectrum of a crystal in the proto-polyp, the mother colony skeleton and of the ECM reveals a prominent calcium peak (Fig. 3). In addition to the calcium peak, a second prominent sulfur peak can be seen in the crystal deposit in the proto-polyp (Fig. 3a). However, sulfur peaks were not observed on the cell section of the proto-polyp (Fig. 3b), but rather peaks corresponding to nitrogen, carbon and oxygen that are associated with the organic matrix were detected. As the initial crystals emerged from the ECM, the sulfur peak observed in those crystals, and not in the mother colony skeleton, may result from relatively abundant sulfate-bearing organic compounds at centers of calcification but lower organic matter concentrations in the bulk skeleton, as suggested by Cuif et al. [33]. These results support the Cuif et al. [33] proposed model of crystal growth that involves a step-by-step growth of aragonite fibers where each step is initiated and guided by a sulfated organic matrix sheet.

### ECM composition

The total ECM was separated by centrifugation into soluble and insoluble fractions. Total aspartic acid plus asparagine content (Asx), measured by HPLC, was ∼20% of total ECM skeletal protein content for both fractions. The second most abundant amino acid was glutamic acid plus glutamine (Glx), also accounting for ∼20% of the total. Thus, these two sets of amino acids account for ∼40% of the total in both soluble and insoluble ECM fractions both in the mother colony and cell culture ECM (Table 1). Similar to our study, Young [15] reported 12–23% of Asx in skeletons from 14 different species of scleractinian corals. Specific functions of the soluble and insoluble ECM fractions in the calcification processes have yet to be determined. While some differences have been found between the two fractions, many similarities have been indicated, including polar amino acid content and protein size and activity [34], [35]. Differences between the two fractions may be ascribed to their roles in either structural framework (insoluble ECM) or crystal nucleation (soluble ECM) [36]–[38]. However, as only one ECM protein has been fully sequenced, the distinctive and/or overlapping nature of soluble and insoluble ECM proteins remains to be seen [39].

**Table 1:** Relative amino acid composition of skeletal and cell culture ECM as determined by HPLC fluorometric analysis.

	Skeleton		Cell Culture	
	Soluble ECM mole %	Insoluble ECM mol %	Soluble ECM mole %	Insoluble ECM mol %
**Asparagine or aspartic acid (Asx)**	19	21	20	27
**Glutamic acid or glutamine (Glx)**	15	18	21	19
**Serine**	9	10	3	4
**Threonine**	20	11	2	1
**Glycine**	18	14	15	10
**Histidine**	5	<1	<1	<1
**Arginine+Alanin**	5	6	8	8
**Tyrosine**	<1	2	2	<1
**Valine**	3	8	6	8
**Methionine**	1	<1	2	2
**Phenylalanine+Isoleucine**	2	6	8	8
**Leucine**	3	4	13	13
**Total**	100	100	100	100

Cell culture ECM is derived from the NaOH-treated pellet.

Sulfur has been shown to be integral to centers of calcification in coral skeletons [33]. We have confirmed a lower amount of sulfur containing material in ECM compared to primary crystals by EDS (Fig. 3). In addition, we measured 0.5–1% methionine residues in *S. pistillata* mother colonies by HLPC (this method does not allow quantification of cysteine).

### Final Remarks

**Figure 5:**
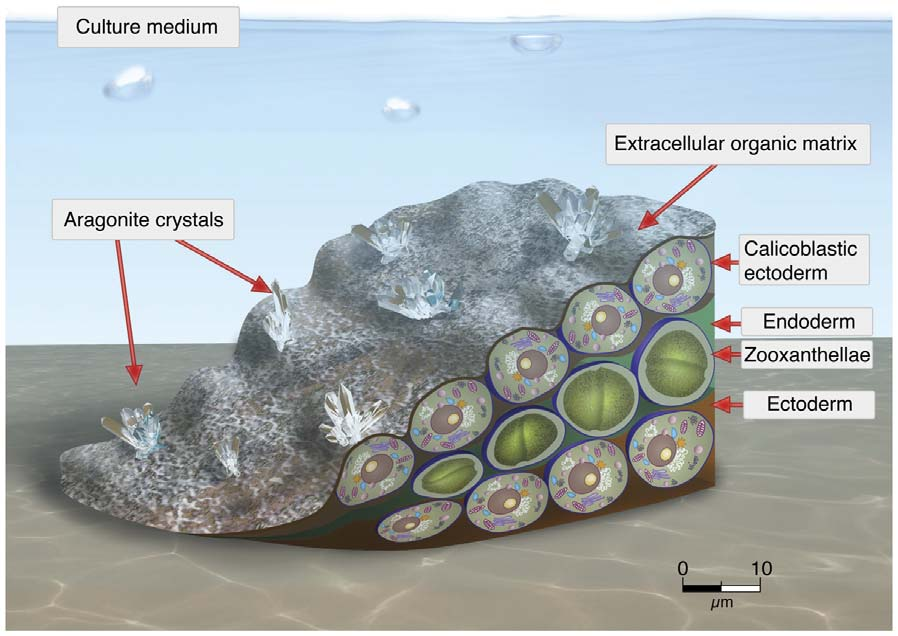
Drawing of a *proto-polyp* showing the three cell layers organization. The lower layer, the ectoderm that is attached to the plate, is covered by the endoderm containing *Symbiodinium* sp. cells, which in turn is covered by the upper layer of calicoblastic cells as seen in Fig. 2B. The calicoblastic layer secretes ECM as seen in Fig. 2C. Aragonite crystals form on top of the ECM (Fig. 3A and C).

In this study we demonstrate that a primary culture of disaggregated coral cells can re-assemble into *proto-polyps* from primary, differentiated cells. Each *proto-polyp* contains three cell layers: a basal layer of ectodermal cells that adheres to the plate is covered with a second layer of endodermal cells containing zooxanthellae (Fig. 5). The top layer contains ectodermal calicoblastic cells exposed directly to medium. In coral colonies aragonite is precipitated on ECM scaffolding that is secreted into the calicoblastic space by ectodermal cells facing the skeleton [2]. In contrast, in our culture the calicoblastic cell-layer is exposed to the artificial calcicoblastic fluid surrounding the *proto-polyp* (Fig. 5). Under these culture conditions, we propose that the calicoblastic cell layer secretes ECM with sulfate rich calcifying centers [33] onto which the aragonite crystals precipitate (Fig. 3). We note that the ECM amino-acid composition produced by the cell culture is similar to that of the mother colony. Aragonite crystals in cell cultures have similar structure and chemical composition to the mother coral colony skeleton; however, they are substantially larger than those in the mother coral (10 µm vs. 2 µm, respectively). The difference is likely due to the fact that in the culture the new skeleton formation is not constricted by the small calicoblatic space as it is inside a coral colony, therefore crystals extend to greater lengths and are lower in density (Fig. 3). Regardless of morphological differences, however, our results clearly suggest that coral cells can catalyze carbonate crystal formation under ambient concentrations of DIC. How that process occurs remains fundamentally unknown. This study demonstrates the potential of coral cell culture for studying physiological mechanisms, including calcification and cell differentiation, at the cellular level. This will provide a critical tool for mechanistically understanding how shifts in ocean pH will impact calcifying corals.

## Supporting Information

Figure S1 **DNA partial sequence.** *S. pistillata* mitochondrion sequence (*Upper*) and 18S rDNA of *Symbiodinium* sp. (*Lower*).
(PDF)

Figure S2 **Relief contrast video of coral cell cultures.** Link to a movie of *proto-polyp* assembly after 72 h in culture medium.
(DOCX)

Figure S3 **Frame by frame confocal imaging:** Frame every 2 µm of Z-stack of a *proto-polyp* at 12 d. The *Symbiodinium* sp. cells are seen by chlorophyll florescence located in the middle of the aggregation (20–50 µm) while animal cells are revealed by GFP fluorescence located in the whole aggregate.
(TIF)
