# Supplementary material for: Aragonite Precipitation by “Proto-Polyps” in Coral Cell Cultures
Source: PLoS One. 2012 Apr 13;7(4):e35049. doi: 10.1371/journal.pone.0035049 (PMC3325950; doi:10.1371/journal.pone.0035049)
Supplement: Figure S1 — DNA partial sequence. S. pistillata mitochondrion sequence (Upper) and 18S rDNA of Symbiodinium sp. (Lower). (PDF) [file pone.0035049.s001.pdf]

5’-

TTTTCCACTAATCATAAAGATATCGGTAGTTTGTATCTAATTTTTGGTGGGGGTGCTGG
TTTAATCGGGACGGCGTTTAGTATGCTTATACGACTCGAGCTTTCTGCGCCCGGAGCGAT
GTTAGGAGATGATCATCTTTATAATGTAATTGTTACAGCACATGCTTTTATTATGATTT
TTTTTTTGGTTATGCCCGTTATGATTGGGGGGTTTGGTAATTGATTGGTCCCATTATATA
TTGGGGCGCCGGATATGGCGTTTCCCCGACTAAACAATATTAGTTTTTGACTTTTGCCCC
CTGCGCTTTTTTTATTATTAGGCTCTGCTTTTATTGAACAAGGGGCGGGGACGGGGTGAA
CAGTTTATCCTCCTCTTGCTAGTATTCAAGCACACTCCGGAGGTTCGGTTGATATGGTTA
TTTTTAGTCTTCATTTAGCTGGGGTTTCTTCTATTTTAGGTGCTATAAACTTTATTACTA
CAATTTTAAATATGCGAGCCCCGGGTGTGTCTTTTAATAAACTACCTTTATTTGTTTGAT
CTATTTTAATAACAGCTTTTTTATTGCTTTTATCTTTACCTGTTTTAGCTGGTGCTATTA
CTATGTTGTTAACAGATAGAAACTTTAATACGACTTTTTTCGATCCAGCGGGTGGCGGG
GACCCAATATTATTTCAGCATCTATTTTGATTCTTTGGGCATCCAGAAGTTTATATTTTA
ATTTTGCCTGGTTTTGGTATGATTTCTCAAATAATCCCGACTTTTGTTGCTAAAAAACAA
GTTTTCGGGTATTTAGGAATGGTTTATGCCATGCTTTCTATTGGAGTATTGGGCTTTACT
GTGTGGGCA -3’

5’-

GCAGTTATAATTTATTTGATGGTTGCTGCTACATGGATAACTGTGGTAATTCTAGAGCT
AATACATGCATCCAAGCCCGACTTCGCAGAAGGGTTGTGTTTATTAGATACAGAATCAAC
CCAGGTTCCATCTGGTAATGTGGTGATTCATGATCACTTGACGAATCGCGCGGCCTTGCC
GACGATGCGTCATTCAAGTTTCTGACCTATCAGCTTCCGACGGTAGTGTATTGGCCTACC
GTGGCAATGACGGGTAACGGAGAATTAGGGTTTGATTCCGGAGAGGGAGCCTGAGAAAT
GGCTACCACATCTAAGGAAGGCAGCAGGCGCGCAAATTACCCAATCCTGACGCAGGGAGG
TAGTGACAAGAAATAACAATACAGGGCATCCATGTCTTGTAATTGGAATGAGTAGAACA
CAAATATCTTTATGAGTATCAATTGGAGGGCAAGTCTGGTGCCAGCAGCCGCGGTAATTC
CAGCTCCAATAGCGTATATTAAAGTTGTTGCGGTTAAAAAGCTCGTAGTTGGAGTTCTG
TTGAGGATGTCCGGTCCGCCCTCTGGATGTGCATCTGGCTCAGCCTTGACATCTTCCCGA
AGAACGTATCTGCACTTGACTGTGTGGTGCGGTATTTGGGACATTTACCTTGAGGAATT

AGAGTGTTTCAAGCAAGCATCCGCTTTGAATACGTTAGCATGGAATAATAAGACAGGAC
CTCAGTTCTATCTTGTTGGTTTCTAGAGCTGANGTAATGATTGATAGGGATAGTTGGGG
GCATTTGTATTTAACTGTCAGANGTGA-3’

Figure S1.
