# Supplementary material for: Aragonite Precipitation by “Proto-Polyps” in Coral Cell Cultures
Source: PLoS One. 2012 Apr 13;7(4):e35049. doi: 10.1371/journal.pone.0035049 (PMC3325950; doi:10.1371/journal.pone.0035049)
Supplement: Figure S2 — Relief contrast video of coral cell cultures. Link to a movie of proto-polyp assembly after 72 h in culture medium. (DOCX) [file pone.0035049.s002.docx]

<ftp://ftp.marine.rutgers.edu/pub/cool/video/2011/>

Name of the file is "aggregation_movie_pic_every_15min.mov"
