# Supplementary figures and images for: Aragonite Precipitation by “Proto-Polyps” in Coral Cell Cultures
Source: PLoS One. 2012 Apr 13;7(4):e35049. doi: 10.1371/journal.pone.0035049 (PMC3325950; doi:10.1371/journal.pone.0035049)

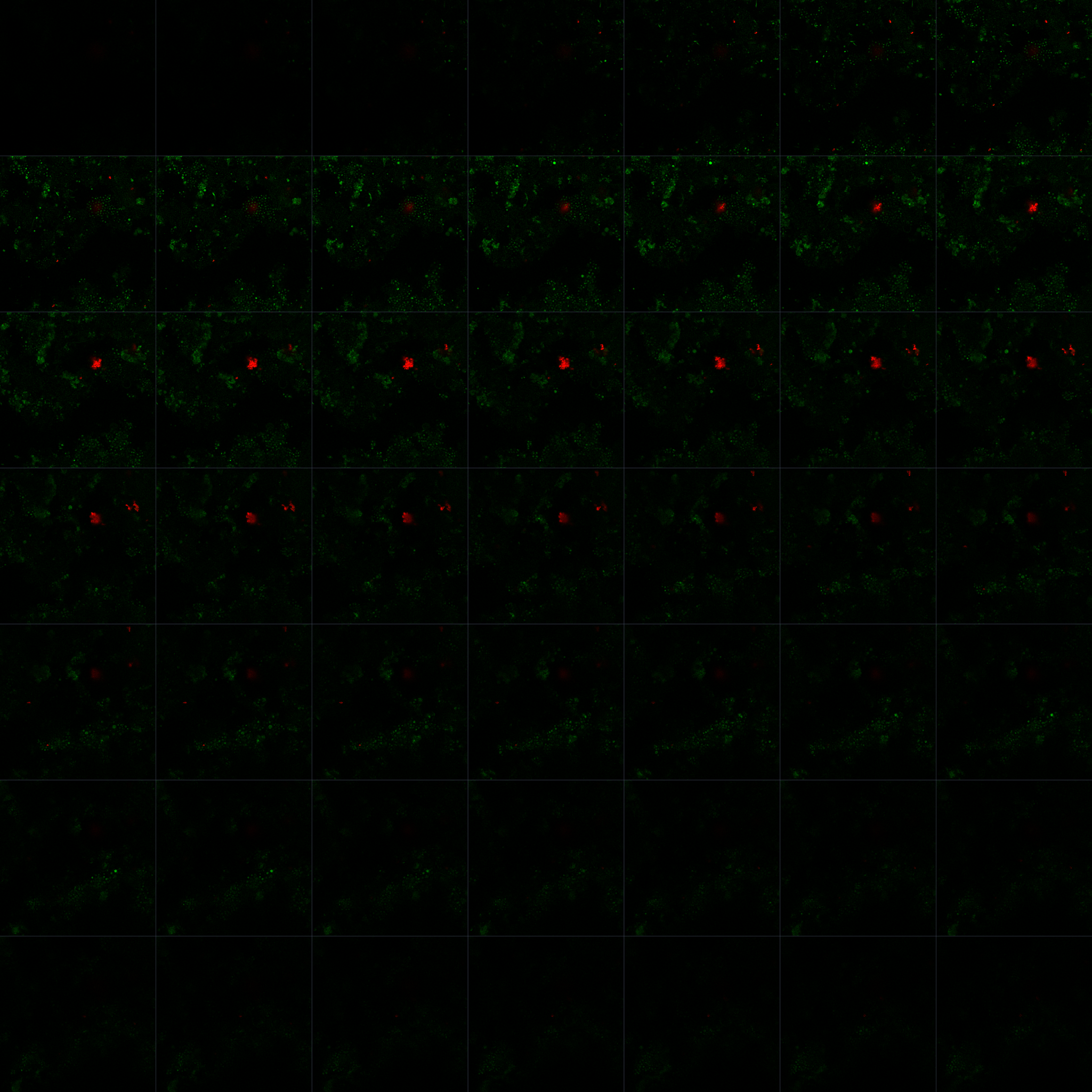

Supplement: Figure S3 — Frame by frame confocal imaging: Frame every 2 µm of Z-stack of a proto-polyp at 12 d. The Symbiodinium sp. cells are seen by chlorophyll florescence located in the middle of the aggregation (20–50 µm) while animal cells are revealed by GFP fluorescence located in the whole aggregate. (TIF) [file pone.0035049.s003.tif]
